# Predictive constitutive modelling of arteries by deep learning

**DOI:** 10.1098/rsif.2021.0411

**Published:** 2021-09-08

**Authors:** Gerhard A. Holzapfel, Kevin Linka, Selda Sherifova, Christian J. Cyron

**Affiliations:** ^1^ Institute of Biomechanics, Graz University of Technology, Stremayrgasse 16/2, 8010 Graz, Austria; ^2^ Department of Structural Engineering, Norwegian University of Science and Technology (NTNU), Trondheim, Norway; ^3^ Institute for Continuum and Material Mechanics, Hamburg University of Technology, Eißendorfer Straße 42, 21073 Hamburg, Germany; ^4^ Institute of Material Systems Modeling, Helmholtz-Zentrum Hereon, Max-Planck-Straße 1, 21502 Geesthacht, Germany

**Keywords:** soft biological tissues, constitutive modelling, deep learning, data-driven modelling, hybrid modelling

## Abstract

The constitutive modelling of soft biological tissues has rapidly gained attention over the last 20 years. Current constitutive models can describe the mechanical properties of arterial tissue. Predicting these properties from microstructural information, however, remains an elusive goal. To address this challenge, we are introducing a novel hybrid modelling framework that combines advanced theoretical concepts with deep learning. It uses data from mechanical tests, histological analysis and images from second-harmonic generation. In this first proof of concept study, our hybrid modelling framework is trained with data from 27 tissue samples only. Even such a small amount of data is sufficient to be able to predict the stress–stretch curves of tissue samples with a median coefficient of determination of *R*^2^ = 0.97 from microstructural information, as long as one limits the scope to tissue samples whose mechanical properties remain in the range commonly encountered. This finding suggests that deep learning could have a transformative impact on the way we model the constitutive properties of soft biological tissues.

## 1. Motivation

Over the last decades, soft tissue biomechanics has substantially contributed to our understanding of various cardiovascular diseases such as aneurysms and dissections. It can help develop improved therapies and intervention strategies by computational simulations. However, realistic and ideally patient-specific simulations require reliable constitutive models that capture the complex and unique mechanical behaviour of soft biological tissues. The mechanical behaviour of the arterial wall is determined by its constituents. For example, the tissue anisotropy is closely related to the local dispersion of collagen fibres. In addition, the waviness and cross-linking of collagen fibres affect the stiffening of arterial tissue under tensile loading. While some phenomena such as fibre dispersion can well be captured with advanced microstructure-based constitutive models [1], the incorporation of more subtle microstructural and histological information for the purpose of predictive constitutive modelling of arterial tissue remains elusive to date. To overcome this limitation, a change of paradigm may be helpful.

Over the past decade, machine learning has attracted rapidly increasing attention in computer vision in general [2], and in medical imaging in particular [3,4]. Deep learning (DL)—a subset of machine learning—has become increasingly popular due to improved computer performance and the availability of tools that make it easy to use. In the life sciences in particular, a large number of innovative DL methods have recently been proposed [5,6] in order to move the technique further and further beyond traditional image analysis.

In soft tissue biomechanics, DL has recently been used to estimate material properties from organ scale *in silico* simulations of physiologically loaded organ geometries, e.g. from abdominal aortic aneurysms [7] or the aorta [8]. Other approaches have focused on developing surrogate models based on machine learning that can complement or replace more expensive finite-element simulations on the organ scale, e.g. to predict the left ventricular mechanics in real time [9]. A first attempt to predict the constitutive properties of soft tissue on the basis of microstructural data is documented in [10]. It was based on an end-to-end DL model that was trained to capture the relationship between raw second-harmonic generation (SHG) images and stress–stretch curves of tissue patches. However, despite some very promising results, the overall accuracy of the predictions of the mechanical properties that the trained deep neural network could make from given image data remained relatively poor. The reason for this may be that standard end-to-end neural networks do not have any prior information about the physical phenomenon to be investigated. As a result, large amounts of data are required to achieve satisfactory predictive capabilities. Unfortunately, in soft tissue biomechanics, such huge amounts of data are often not available due to the significant costs typically associated with experiments. To overcome such problems, physics-informed machine learning methods have recently been proposed for constitutive modelling [11]. These can achieve a high level of predictive accuracy from a surprisingly small set of training data, since their architecture has physical knowledge that enables them to use the available training data with the utmost efficiency. However, such concepts have not yet been systematically applied in soft tissue biomechanics.

With this in mind, the present study focuses on the following key question. Suppose we have a number of tissue samples for which mechanical, histological and microstructural imaging data are available. How can we predict the mechanical response of a new, unknown tissue sample based solely on its histological and microstructural imaging data? To answer this question, we introduce a novel hybrid modelling framework that combines advanced theory-based constitutive modelling [12] with DL. A deep neural network is used to learn the parameters of the continuum mechanical constitutive law for arterial tissue introduced in [12] from SHG imaging data and histological data. Of course, when compared with methods that rely entirely on DL (i.e. end-to-end DL models), our hybrid modelling framework naturally ensures a physically reasonable output and requires much less training data to achieve acceptable accuracy in predicting mechanical properties from imaging and histological data.

Our paper is structured as follows. In §2, we briefly describe the employed experimental data published previously [13]. In §3, we summarize the constitutive model documented in [12], while in §4 we present a novel hybrid modelling (HM) framework. In addition, we validate its predictive capabilities and show that it significantly surpasses the standard curve fitting. Finally, in §5, we summarize the proposed HM framework and discuss its current limitations as well as future directions.

## 2. Experimental data processing

Overall, three different types of data published in [13] form the basis of our work in this article: Cauchy stress–stretch data from mechanical tests, volume fractions from histological analyses and SHG images.

### 2.1. Mechanical data

Circumferential and axially oriented dog bone samples were taken from healthy and aneurysmatic human aortic medias, preconditioned to 50 kPa (engineering stress) in 5 loading–unloading cycles and subjected to uniaxial extension tests to failure at a crosshead speed of 2 mm min^−1^. Smooth muscle cells were assumed to be relaxed during testing as they were reported to have largely lost their functionality due to the freezing and subsequent thawing that all our tissue samples were subjected to [13]. Using the continuously recorded force and displacement measurements of the test, as well as thickness and width measurements prior to the start of the test, Cauchy stress and stretch data were obtained. Then the mechanical data were manually cut off at stretches where either jumps or softening in the curves were observed (for a detailed discussion see [13]).

### 2.2. Histological data

After a successful test in which the sample ruptured in the gauge region, the two halves of the sample were structurally fixed in 4% formaldehyde solution. One half was subjected to histological analysis using hematoxylin, eosin and Elastica van Gieson staining to quantify the volume fractions of collagen (CO), elastic fibre (EF) and smooth muscle cell (SMC), hereinafter referred to as *ϕ*_CO_, *ϕ*_EF_ and *ϕ*_SMC_, respectively.

### 2.3. Microstructural data

The second half of the ruptured tissue samples was optically cleared with a solution of benzyl alcohol to benzyl benzoate to generate SHG, as described in [13,14]. The collagen fibre orientation in the circumferential–axial (in-plane) and the radial–axial (out-of-plane) planes was extracted from SHG images and used to obtain the mean fibre angle *α* (definition see §3), the in-plane dispersion parameter *κ*_ip_, and the out-of-plane dispersion parameter *κ*_op_ according to their definitions in [12]. In addition, intensity plots were created for each sample, showing the change in collagen fibre orientation over the sample thickness, using the data extracted from in-plane images.

**Figure 1.**
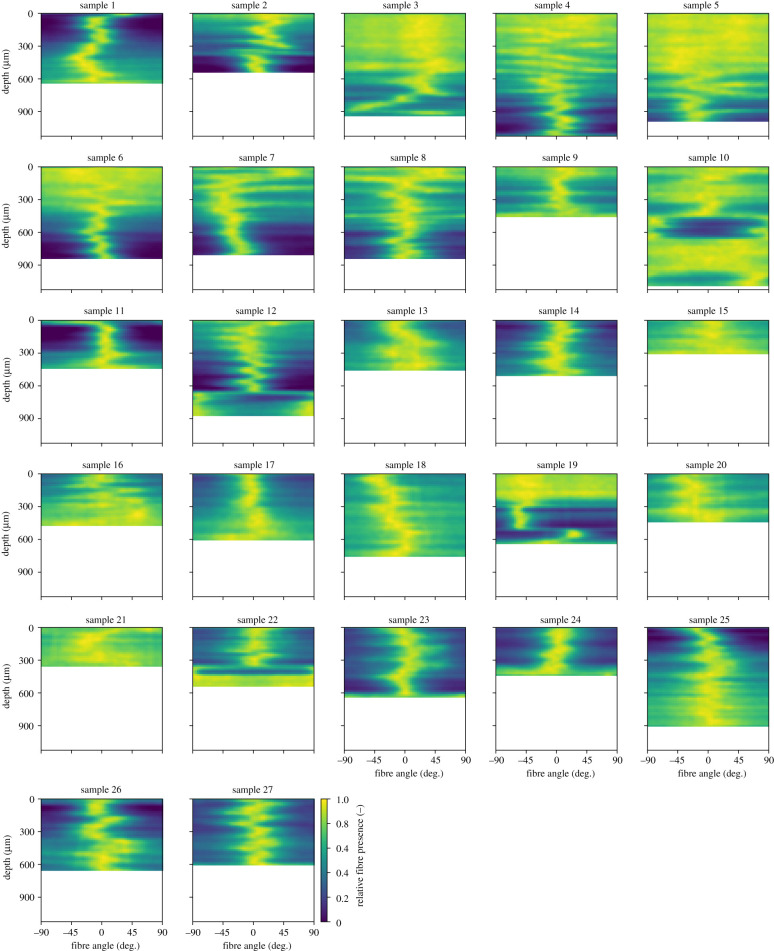
Intensity plots showing the orientation and dispersion of the collagen fibres over the thickness of the tissue samples examined in [13]: the plots were obtained from SHG images and prepared for use in DL by rescaling. Yellow and dark blue colours indicate the presence and absence of fibres for the entire range of possible fibre angles (relative to the circumferential tissue direction), respectively.

All images were processed in the same way in three steps before they were used as training data for DL in the form shown in figure 1. First, all intensity plots were rescaled such that the scale of the pixels was identical for all the plots and the thickest sample covered 68 pixels in the direction of the thickness (vertical). Accordingly, thinner samples covered fewer pixels in the thickness direction. In the angular (horizontal) direction, all samples were scaled to 54 pixels. Second, linear interpolation scaled all images to 68 pixels in the thickness (vertical) direction to achieve a uniform image format suitable for DL. Third, data augmentation was performed by taking additional samples by applying various random periodic shifts to the image data in the angular (horizontal) direction.

## 3. Theory-based constitutive modelling

### 3.1. Constitutive model

Here, we model soft biological tissues as mechanical bodies. According to the theory of nonlinear continuum mechanics, the deformation of these bodies is described by a reference configuration (e.g. the configuration before the deformation) and a current configuration (after the deformation). The reference configuration consists of material points X that are mapped by the deformation to their current position χ(X). Locally, the deformation can be characterized by the so-called deformation gradient F=∂χ/∂X, while F can be used to define the right Cauchy–Green tensor $C=F^{T}F$ and the left Cauchy–Green tensor $b=FF^{T}$. We model the constitutive behaviour of arterial tissues following the approach introduced in [12]. Thereby, we include two symmetric collagen fibre families with the two corresponding mean fibre directions

**Figure 2.**
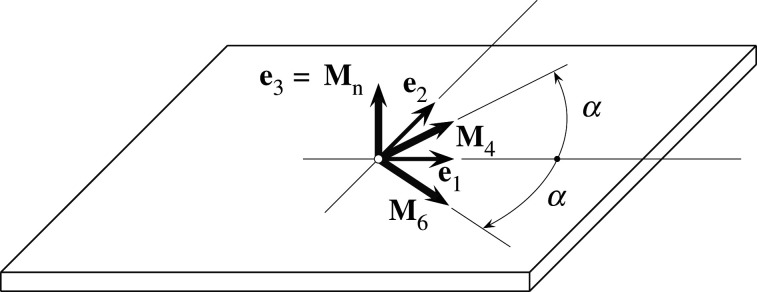
Schematic of arterial tissue sample: two fibre families are represented by their respective mean directions $M_{4}$ and $M_{6}$ in the $(e_{1}$–$e_{2})$ (circumferential–axial) plane, each with an angle *α* relative to the circumferential direction $e_{1}$ of the arterial tissue. The normal direction $e_{3}$ (thickness direction of the arterial wall) is referred to as $M_{n}$ (adapted from [12]).

3.1 $$\begin{array}{rl} M_{4} & =\cos\alpha e_{1}+\sin\alpha e_{2}\;\text{and}\\ M_{6} & =\cos\alpha e_{1}-\sin\alpha e_{2}, \end{array}$$

where *α* is the mean fibre angle in the $\left(e_{1}\text{–}e_{2}\right)$ plane with the circumferential direction $e_{1}$ and the axial direction $e_{2}$; see figure 2. Now, we can comfortably describe the continuum deformation with the set of invariants

3.2 $$\begin{array}{l} I_{1}=\text{tr}C\text{,}\;\;I_{i}=C\;:\;M_{i}⊗M_{i}\text{,}\;\;i=4\text{,}\;6,\\ \;\;\;I_{\text{n}}=C\;:\;M_{\text{n}}⊗M_{\text{n}}\text{,}\; \end{array}$$

where $M_{n}$ denotes the out-of-plane unit direction vector (normal to the circumferential–axial plane of the arterial tissue). To capture the anisotropic mechanical behaviour governed mainly by the alignment of collagen fibres, we employ the so-called generalized structure tensor

3.3 $$\begin{array}{l} H_{i}=AI+BM_{i}⊗M_{i}+(1-3A-B)M_{\text{n}}⊗M_{\text{n}},\\ \;\;i=4,\;6. \end{array}$$

where *κ*_ip_ and *κ*_op_ quantify the in-plane and out-of-plane collagen dispersions, respectively. By assuming an additive decomposition of the strain-energy function Ψ into the ground matrix contribution $\Psi_{m}$ and the collagen fibre contribution $\Psi_{f}$, the resulting Ψ can be given as [12]

3.5 $$\Psi =\Psi_{m}(C)+\sum_{i=4,6}\Psi_{\;f,i}(C,H_{i}).$$

We model the ground matrix as a neo-Hookean material, while the fibre contribution is of exponential type, i.e.

3.6 $$\begin{array}{rl} \Psi_{m} & =\frac{c}{2}(I_{1}-3),\\ \Psi_{\;f,i} & =\sum_{i=4,6}\frac{k_{1}}{2\;k_{2}}\{\exp[k_{2}{\left(I_{i}^{*}-1\right)}^{2}]-1\}, \end{array}$$

where *c*, *k*_1_ > 0 are stress-like material parameters, *k*_2_ is a dimensionless material parameter, and *I*_i_* is the generalized invariant, i.e.

3.7 $$I_{i}^{*}=\mathrm{tr}(CH_{i})=A\;I_{1}+B\;I_{i}=(1-A-B)\;I_{n},\;i=4,6.$$

Modelling arterial tissue as an incompressible hyperelastic material, the second Piola–Kirchhoff stress tensor S can then be computed as

3.8 $$S=2\left(\frac{\partial \Psi }{\partial I_{1}}I+\sum_{i=4,6}\frac{\partial \Psi }{\partial I_{i}}M_{i}⊗M_{i}+\frac{\partial \Psi }{\partial I_{n}}M_{n}⊗M_{n}\right)-pC^{-1},$$

where *p* denotes a Lagrange multiplier (pressure) to enforce incompressibility.

### 3.2. Parameter fitting to experimental data

The constitutive model has six parameters in total. The three structural parameters *κ*_ip_, *κ*_op_, *α* can be determined from the fibre orientation and in-plane dispersion information carried by the intensity plots in figure 1. By contrast, the three material parameters *c*, *k*_1_, *k*_2_ remain *a priori* unknown. The classical way to determine these is curve fitting, using data from mechanical testing. In uniaxial extension tests, the three principal stretches *λ*_1_, *λ*_2_ and *λ*_3_ in circumferential, axial and thickness direction define the deformation gradient as a diagonal tensor, here written in matrix form as $\left[F]=\mathrm{diag}[\lambda_{1},\lambda_{2},\lambda_{3}\right]$. Combining $\sigma ={FSF}^{T}$ with (3.8) yields the non-zero components of the Cauchy stress tensor σ as

3.9 $$\begin{array}{rl} \sigma_{11} & =[c+4(A+B\cos^{2}\alpha )\psi_{4}^{\prime }]\;\lambda_{1}^{2}-p,\\ \sigma_{22} & =[c+4(A+B\sin^{2}\alpha )\psi_{4}^{\prime }]\;\lambda_{2}^{2}-p,\\ \sigma_{33} & =[c+4(1-2A-B)\psi_{4}^{\prime }]\;\lambda_{3}^{2}-p,\\ \mathrm{with}\;\psi_{4}^{\prime } & =k_{1}(I_{4}^{*}-1)\exp[k_{2}{\left(I_{i}^{*}-1\right)}^{2}]. \end{array}\}$$

Incompressibility of the tissue requires *λ*_1_*λ*_2_*λ*_3_ = 1. Moreover, only *σ*_*jj*_ is non-zero in a uniaxial test with loading in the *j*-th direction. That is, for a circumferential test we have *j* = 1 and *σ*_22_ = *σ*_33_ = 0, for an axial test we have *j* = 2 and *σ*_11_ = *σ*_33_ = 0.

With these equations, the unknown material parameters can be expressed as experimentally measured variables. The material parameters can then be determined by curve fitting using the experimentally measured stress–stretch curves. For this purpose, the least-squares method implemented in the LMFIT package, Python 3.7 [15], was used. First, the material parameters were individually fitted for each tissue sample. In a second step, this information was used for predictions. That is, a specific sample of interest has been selected. For this sample, the structural parameters *κ*_ip_, *κ*_op_, *α* were calculated directly from the imaging data. The remaining constitutive parameters were set equal to the median values across all other samples. This predictive protocol was repeated by running the sample of interest (for which predictions were made) through the entire set of available samples. This approach is also known as leave-one-out cross-validation (LOO-CV) and was performed separately for each sample in the dataset (i.e. validation fold). That is, each sample was considered a sample of interest once. To quantify the goodness of fits, we used the coefficient of determination *R*^2^, i.e.

3.10 $$R^{2}=1-S_{\mathrm{res}}/S_{\mathrm{tot}},$$

with

3.11 $$S_{\mathrm{res}}=\sum_{i}{\left(\sigma_{\exp}^{i}-\sigma^{i}\right)}^{2},\;S_{\mathrm{tot}}=\sum_{i}{\left(\sigma_{\exp}^{i}-{\bar{\sigma }}_{\exp}\right)}^{2}.$$

Here, the index *i* runs through all contained experimental data points. Each *i* is associated with a certain uniaxial stretch *λ*^*i*^ (either in the axial or in the circumferential direction of the tissue patch), a related experimentally measured Cauchy stress *σ*_exp_ in the same direction and a Cauchy stress component *σ*^*i*^ also in this direction and calculated from our constitutive model, i.e. (3.9). The mean values of the experimental stress values contained is ${\bar{\sigma }}_{\exp}$.

The values of *R*^2^ found in the LOO-CV method described above characterize the accuracy with which the constitutive properties of a new (mechanically not yet tested) tissue sample can be predicted using an SHG image-based computation of the structural parameters *κ*_ip_, *κ*_op_, *α* with standard curve fitting for the remaining material parameters. This accuracy marks the current state of the art, and the method for achieving this is referred to below only as the standard fitting. Remarkably, the above method consistently resulted in higher predictive accuracies than an alternative method in which we determined *c*, *k*_1_, *k*_2_ by fitting all of the stress–stretch curves simultaneously except for those of the sample of interest. Therefore, the latter method is not discussed further herein.

Predictions using the standard fitting method described above showed poor to moderate quality (median *R*^2^ = 0.631, s.d. ±0.46) when samples with *R*^2^ ≤ −1 were excluded, and were of poor quality (median *R*^2^ = 0.14, s.d. ±5 × 10^8^) if all samples were taken into account. In 8 out of 27 cases (37%), *R*^2^ was very low (i.e. *R*^2^ ≤ −1); see also figure 6, samples 4, 6, 8, 13, 14, 20, 25 and 27.

## 4. Hybrid modelling framework

In this section, we present a novel hybrid model (HM) that combines DL and theory-based modelling with the aim of predictive constitutive modelling of arteries. Our HM uses the theory-based constitutive model that was introduced in [12] and summarized above in §3.1. In relation to this model, it uses DL to determine the *a priori* unknown material parameters *c*, *k*_1_ and *k*_2_. In this way, our HM enjoys the advantages of machine learning, but includes the essential theoretical prior knowledge that is summarized in the theory-based model introduced in [12]. It is expected to reduce considerably the amount of training data required to learn the stress–stretch relationship for a given tissue sample, compared to standard end-to-end DL models that involve a deep neural network used to learn the entire constitutive equation from scratch.

### 4.1. Architecture

**Figure 3.**
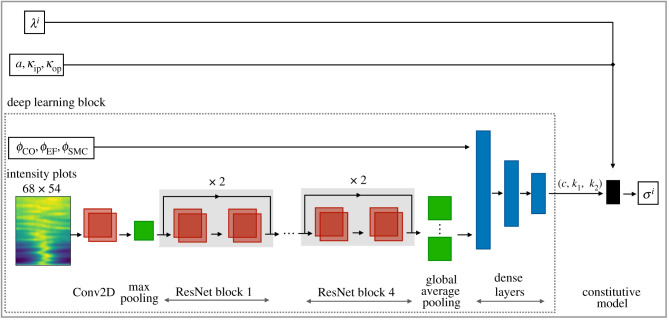
Hybrid model architecture: deep learning block calculated from the intensity plots and histological data (collagen: *ϕ*_CO_, elastic fibres: *ϕ*_EF_, smooth muscle cells: *ϕ*_SMC_) the material parameters (*c*, *k*_1_, *k*_2_). Together with the structural parameters (*α*, *κ*_ip_, *κ*_op_), which can be calculated directly from the intensity plots except for the latter. This yields a constitutive law that maps stretches *λ*^*i*^ to Cauchy stresses *σ*^*i*^ (in the same direction).

The HM architecture consists of two parts. One is a deep neural network model referred to as DL block and the other is the theory-based constitutive model, as summarized in §3. The DL block maps the intensity plots and the histology data (*ϕ*_EF_, *ϕ*_CO_, *ϕ*_SMC_) as input of a deep neural network to the material parameters (*c*, *k*_1_, *k*_2_). The DL block output (*c*, *k*_1_, *k*_2_) forms together with the structural parameters (*α*, *κ*_ip_, *κ*_op_), which are calculated in the standard way directly from the SHG images, and the stretch data the input of the constitutive relation (3.8), which gives the Cauchy stress. The architecture of our new HM is illustrated in figure 3. The DL block can be trained in such a way that the HM optimally resembles the stress–stretch relationship found in the training data. Once the HM has been trained in this way, it can be used to predict the stress–stretch relationship even for tissue samples for which SHG images and histological data are available but mechanical testing data are not yet available.

For the DL block we used a 17 layer residual network (ResNet) encoder (down-sampling) [16] together with a three-layer, densely connected feedforward neural network (DCFNN). ResNets are widely applied in image classification and benefit from the advanced method of deep residual learning, while DCFNNs are able to approximate any continuous function given by the universal approximation theorem [17]. The residual network consisted of a 7 × 7 two-dimensional convolutional (Conv2D) layer with maximum pooling (3 × 3, stride 2), and 4 consecutive residual blocks, as shown in figure 3. Each residual block contains four Conv2D layers with ReLu activation functions and a batch normalization as well as two residual connections, one after each sequence of two Conv2D layers. The Conv2D layers were initialized with two filters and doubled after each residual block. The global average pooling is applied after the last residual block, and this ResNet output is fed together with the histology data into a DCFNN, which consists of three layers with [12,8,4] neurons and softplus activation functions [18].

**Remark.**

The exact dense layer architecture used in our study was determined by hyperparameter tuning. For this purpose, we have tested different layer architectures with the training and validation strategy 1 described below in §4.2. Finally, we chose the architecture that performed best in this comparative study, following the concept of grid search, as also pointed out in [19].

### 4.2. Model training and validation

Our HM only gains its predictive abilities by training the DL block. During training, the weights of the deep neural network are adjusted in such a way that they optimally resemble the stress–stretch relationship in the training data. We started the training process with the widely used Glorot weight initialization [20]. For the training, we used Adam optimization [21] to adjust the weights so that the mean squared error (MSE) is minimized as a loss function, i.e.

4.1 $$\mathrm{MSE}=\sum_{i}|\sigma^{i}-\sigma_{\exp}^{i}{|}^{2},$$

where *i* runs through all tissue stretches contained in the training data and *σ*^*i*^ and $\sigma_{\exp}^{i}$ are defined analogously to (3.11). The training was carried out in batches, each randomly sampling 32 stress–stretch pairs from the entire training data. MSE minimization was performed by running through these batches and modifying the weights of the neural network after each batch to minimize their respective MSE contribution. As soon as this has been achieved for all batches, a so-called epoch of the training process is finished and the next epoch starts, with all batches being run through again. In total, the training continued for 1000 epochs. The learning rate was fixed at 0.001 during training at various layers of the network. Since the Glorot weight initialization involves a certain randomness, the result of the training is also not deterministic. Therefore, training with different initializations was performed ten times in each scenario and the best (based on its performance when applied to the validation dataset) was selected from the resulting trained networks. Our complete framework was implemented using the open-source software library and the Keras machine intelligence platform with TensorFlow backend [22,23].

In DL, so-called over-fitting is a common limitation. That is, if a network is to learn from given training data for too many epochs, after a certain point in time it may no longer learn general features of the problem of interest, but rather specific details of the training data that were selected at random to inform the network about the problem. To avoid such over-fitting, the available data can be divided into three subsets, the training data, the test data and the validation data. For training purposes only the training data for a certain number of epochs are made available to the network. The network’s predictive performance for the different epochs is then compared using the test data. Typically, one observes that this predictive performance improves in the first epochs (learning stage), while it decreases after a critical epoch (over-fitting stage). By simply selecting the trained state of the network rather than the state after the last epoch, but rather the one at the end of the epoch in which the best performance is observed in the test, the problem of over-fitting can usually be largely avoided. Once the trained state of the network has been determined in this way, its predictive performance is assessed on the samples that make up the so-called validation set and that have been completely withheld by the neural network up to this point, so that the network’s performance on this dataset shows its predictive abilities.

The amount of data available is often decisive to the success of machine learning. Often, when big data are available, about 20% of the data are used as a test set and 20% of the data are used as a validation set, so only 60% are used in the actual training process. Since only small data (27 tissue samples) were available in our case, we had to make sure that the machine learning results were not being influenced too much. To this end, we have combined three strategies for this purpose.

*LOO-CV:* we used a so-called LOO-CV scheme. In such a scheme, only one sample is retained for the validation set. In order to evaluate a representative performance, the LOO-CV contains several passes in which different random samples are retained for validation. At the end, the mean or median performance is calculated in all these runs. LOO-CV minimizes the amount of data that is held back for validation purposes and thus maximizes the amount of data that are available for training purposes. It is therefore particularly helpful when only small data are available, since around 20% of the data do not be retained for validation purposes, as is usually the case.

**Figure 4.**
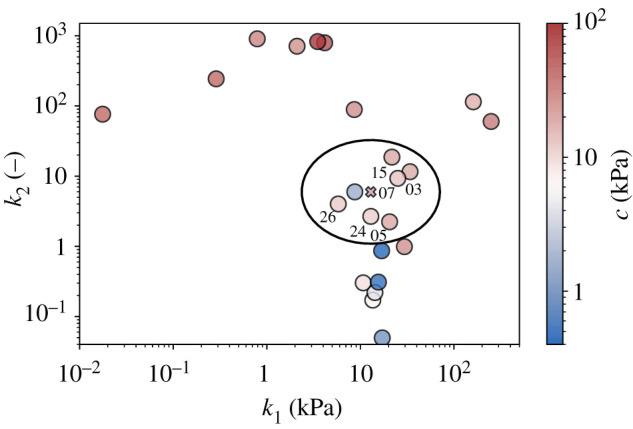
Representation of the latent space (approximated by the *c*–*k*_1_–*k*_2_ parameter space): individual samples are located based on sample-specific fitting of the material parameters to their specific stress–stretch curves. The red cross marks the median point of all samples, while the black ellipse marks a small region of interest around it, where the samples with the most common mechanical properties are located.

*Minimal representative test set:* the smaller the test set, the larger the amount of data that can be used for training. Since LOO-CV can be used to minimize the amount of data retained for validation purposes, other strategies can be used to reduce the amount of data retained for testing purposes. Here, we have only selected a single test sample. However, we did this in such a way that it was particularly representative to compensate for the small size of the test set. For this purpose, we looked at what is known as latent space, the feature space of the data that is used for machine learning. In our case, the latent space can be approximated as the three-dimensional space spanned by the parameters *c*, *k*_1_ and *k*_2_ in the theoretical model described in §3.1. The position of individual samples in this space can be determined by individual curve fitting and is illustrated in figure 4. The median of all samples is illustrated by a red cross. To ensure that our individual test sample was as representative as possible, in our LOO-CV scheme we always selected test sample first that comes closest to the median of the 26 samples not used for validation.

*Confined validation set:* DL is known to be powerful at interpolating, but has difficulty extrapolating from [19]. Therefore, when thinking of the data in latent space, machine learning tends to work best for samples in the centre of the data cloud in that space, while a decreased predictability is often observed for samples near the boundaries of that cloud. With small data, this problem can particularly severe as the relative size of the boundary regions of the data cloud increases. To solve this problem with very small data, we limited the range from which the validation samples in the LOO-CV scheme were selected. Instead of going through all the samples with the validation sample in this scheme, we only went through the samples within a logarithmically scaled sphere around the median point in the latent space, which is referred to below as region of interest (ROI). The radius of this sphere was heuristically defined as 0.74 orders of magnitude. The samples with mechanical properties are collected in the most common range (sample numbers 3, 5, 7, 15, 24 and 26).

### 4.3. Results

**Figure 5.**
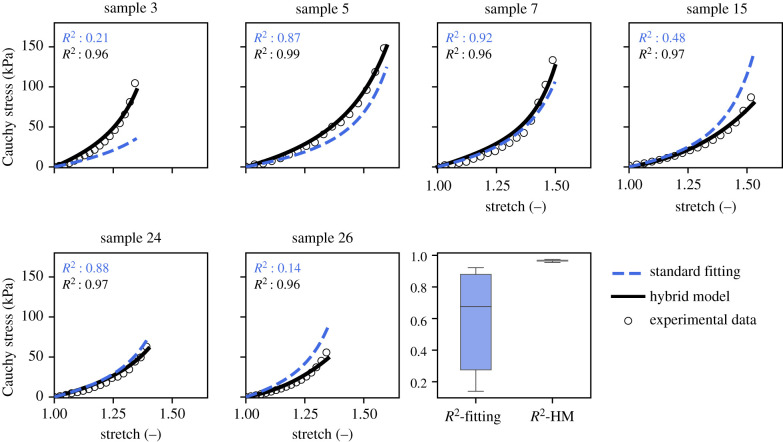
Predicted stress–stretch curves for validation samples in the LOO-CV method with standard fitting (*reference*) compared to our hybrid model and box plot with *R*^2^-statistics.

After training, our hybrid model achieved a predictive accuracy for the validation samples in the ROI with a median of *R*^2^ = 0.966, s.d. ± 0.01. By contrast, the standard LOO-CV fitting, which was also only used for the samples in the ROI, only achieved a median of *R*^2^ = 0.676, s.d. ±0.33 (see also table 2 and figure 5).

**Figure 6.**
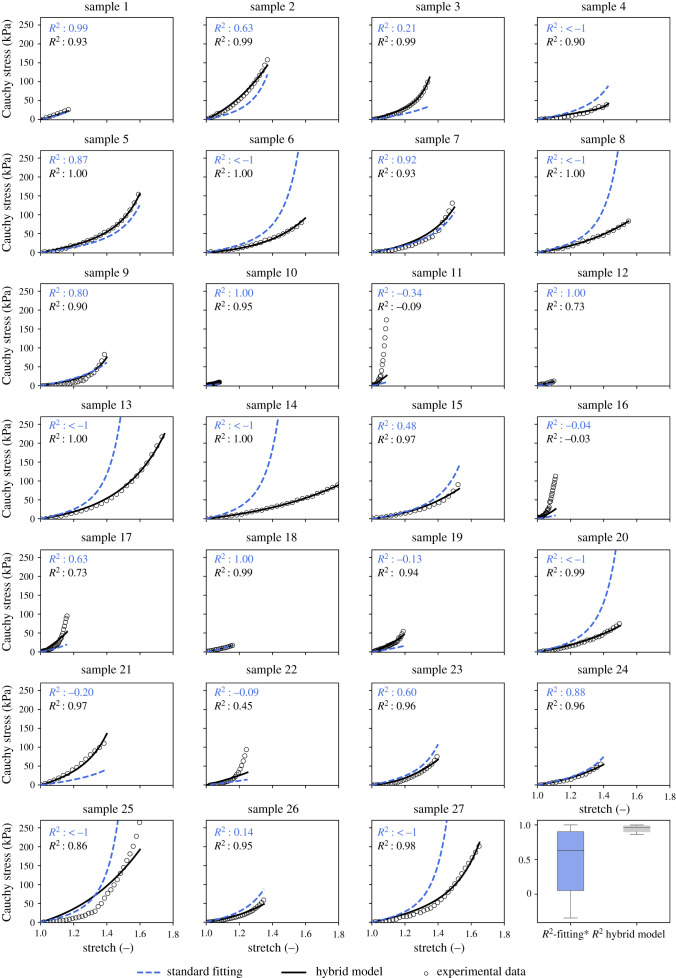
Predicted stress–stretch curves for validation samples in the LOO-CV method with standard fitting compared to our hybrid model (trained with a test sample that corresponds to the validation sample and the LOO-CV scheme passing through all 27 tissue samples) and box plot with *R*^2^-statistics. * means excluding samples with *R*^2^ < −1.

Of course, if both methods are used not only for predictions for samples in the ROI but also for samples with more unusual properties, their predictive accuracy decreases. It is an interesting question whether this decline could be prevented if a larger dataset were available to inform both prediction methods. While an unequivocal answer is impossible in this study, one can at least try to mimic this situation to some extent. To this end, we again applied a LOO-CV scheme. This time we allowed the scheme to run through all of the 27 samples as validation samples. However, to simulate a situation in which more data are available, we also used the validation sample as a test sample for our hybrid model. This mimics a situation where there are enough data available that an amount of test data large enough to be representative of the entire problem can be withheld from the training data. This can trigger an early stop of the learning process after a suitable epoch, even for samples that have rather unusual properties and are far from the most common range. In fact, our hybrid model still achieved a median of *R*^2^ = 0.964, s.d. ± 0.28 with this method. By contrast, the standard LOO-CV fitting, as already mentioned at the end of §3.2, only achieved a median of *R*^2^ = 0.631, s.d. ±0.46, even if the eight worst results have already been skipped, and of only *R*^2^ = 0.140, s.d. ±5 × 10^8^ otherwise; see also table 1 and figure 6. The predictive accuracy of the hybrid model was exceptionally low for only samples 11 and 16 (i.e. *R*^2^ < 0). The standard fit had problems with these samples as well, but in fact even worse and additionally with a number of other samples. These results give hope that a larger database could actually enable our hybrid model to extend its excellent predictive capabilities well beyond the ROI for which they are demonstrated in this article. By contrast, against the background of the above numbers, it seems questionable whether a standard curve fitting scheme could ever achieve similar predictive accuracy, even if it could rely on a much larger database.

**Table 1.** Results of the LOO-CV prediction for the standard fit compared to the hybrid model: predictive accuracy *R*^2^ and obtained material parameters *c*, *k*_1_ and *k*_2_.

	standard fitting				hybrid model			
sample	*R*^2^	*c*	*k*_1_	*k*_2_	*R*^2^	*c*	*k*_1_	*k*_2_
3	0.206	16.42	12.08	2.36	0.963	6.05	34.08	29.32
5	0.869	16.58	12.08	3.04	0.992	1.56	23.99	21.74
7	0.922	19.65	12.33	2.36	0.955	3.49	26.75	9.24
15	0.483	16.42	12.97	2.36	0.974	0.11	10.66	17.02
24	0.883	19.65	12.08	2.85	0.968	1.92	21.08	9.84
26	0.140	19.65	12.97	2.36	0.960	0.00	4.56	16.72
median	0.676	18.11	12.21	2.36	0.966	1.74	22.54	16.87

**Table 2.** Results of LOO-CV prediction for the standard fit compared to the hybrid model (trained with a test sample that corresponds to the validation sample and the LOO-CV scheme passing through all 27 tissue samples): predictive accuracy *R*^2^ and obtained material parameters *c*, *k*_1_ and *k*_2_. * means excluding samples with *R*^2^ < −1.

	standard fitting				hybrid model			
sample	*R*^2^	*c*	*k*_1_	*k*_2_	*R*^2^	*c*	*k*_1_	*k*_2_
1	0.994	16.42	12.97	2.36	0.929	28.25	9.10	0.00
2	0.633	16.42	12.08	3.04	0.986	38.35	33.31	0.00
3	0.206	16.42	12.08	2.36	0.995	45.05	13.75	16.73
4	<−1	19.65	12.08	3.04	0.901	23.81	0.02	19.62
5	0.869	16.58	12.08	3.04	0.998	22.64	17.45	2.42
6	<−1	19.65	12.08	3.04	0.995	6.35	12.56	0.41
7	0.922	19.65	12.33	2.36	0.935	17.19	22.04	1.28
8	<−1	19.65	12.97	3.04	0.998	14.95	10.66	0.00
9	0.798	19.65	12.08	2.36	0.897	17.03	9.75	5.47
10	0.997	16.42	12.97	3.04	0.954	36.55	66.73	0.00
11	−0.345	16.42	12.97	2.36	−0.087	36.87	58.85	0.00
12	0.996	16.42	12.97	2.36	0.731	29.44	28.04	0.00
13	<−1	19.65	12.08	3.04	0.996	12.05	12.34	0.22
14	<−1	19.65	12.97	3.04	0.999	13.96	3.33	0.00
15	0.483	16.42	12.97	2.36	0.965	12.16	16.93	0.00
16	−0.039	16.42	12.97	2.36	−0.029	67.77	50.37	24.01
17	0.631	16.42	12.97	2.36	0.734	30.40	49.28	0.00
18	1	16.42	12.97	2.36	0.991	15.99	13.58	1.13
19	−0.129	16.42	12.97	2.36	0.938	114.86	322.75	10.40
20	<−1	19.65	12.97	3.04	0.986	15.76	9.20	0.00
21	−0.202	16.42	12.08	3.04	0.972	32.67	61.95	3.77
22	−0.094	16.42	12.08	2.36	0.447	38.31	63.03	0.00
23	0.603	19.65	12.08	3.04	0.964	2.54	24.20	0.00
24	0.883	19.65	12.08	2.85	0.964	13.80	17.05	0.00
25	<−1	19.65	12.08	3.04	0.862	27.15	19.75	0.00
26	0.14	19.65	12.97	2.36	0.951	7.16	15.31	0.00
27	<−1	19.65	12.08	3.04	0.984	22.42	7.61	0.64
median	0.631*	16.58	12.33	2.85	0.964	22.64	17.05	0.00

## 5. Conclusions

Significant advances have been made in constitutive modelling of soft biological tissues in the last decades. The methods proposed so far can nowadays describe the mechanical behaviour of such tissues almost perfectly, but they largely lack the ability to predict it from information about the tissue composition and the microstructure. The authors as well as others have tried to make such predictions with classical methods such as linear regression; however, so far only with limited success. In this article, we would like to help fill this gap. We have proposed a novel hybrid model that combines advanced theoretical modelling according to [12] with machine learning. We have demonstrated that this hybrid model can predict the constitutive behaviour (i.e. the stress–stretch curve) of soft biological tissues based on information about the tissue composition (histological data) and microstructure (SHG images). The predictive accuracy reaches a median of *R*^2^ = 0.966, s.d. ± 0.01 for samples whose mechanical properties fall into the most frequently encountered regime. Outside this regime, the predictive accuracy decreases. However, further analysis at least gives hope that this decline could be stopped if more data were available to train our deep neural network. More studies, based on more data, are needed to confirm or deny this hope.

It is worth highlighting that the predictive accuracy of our hybrid model was far better than that of traditional techniques used to make sample-specific predictions by simply fitting the material parameters that cannot be calculated directly from SHG images. Even when applied only to tissue samples with properties in the most common range, such conventional techniques allow predictions of the stress–stretch curves only with a median of *R*^2^ = 0.676, s.d. ± 0.26. In addition, further extension of the prediction range to include samples with more unusual properties resulted in a substantial decrease in the accuracy of the predictions.

This clearly demonstrates the superior predictive capabilities of our novel hybrid constitutive model, which enables predictions of the mechanical properties of soft biological tissues with unprecedented accuracy. In particular, this suggests that machine learning may be able to discover relevant information in experimental data that has so far passed the attention of people working on soft tissue modelling. This once again confirms the transformative effects that machine learning can also have on biomechanics.

A key element of our hybrid model is the theoretical prior knowledge that it inherits due to its architecture from the advanced theoretical constitutive model introduced in [12]. This prior knowledge helps to significantly reduce the amount of training data required, and enables highly accurate predictions based on a sample count as low as 27. In particular, a previous attempt [10] to predict the constitutive properties of soft tissues using a standard end-to-end DL architecture gave relatively poor prediction accuracy, even though almost twice the amount of data was used.

Hybrid modelling cannot only help reduce the amount of training data required. An additional advantage is a constitutive model in a classical form that can easily be used in any computational (e.g. finite-element) simulation. It can also be hoped that the prior knowledge in our framework protects against over-fitting and improves the robustness against errors in the datasets as well as the ability to extrapolate. These advantages have not yet explicitly been shown here, but were recently demonstrated for another type of hybrid constitutive model described in [11]. All of this clearly shows the critical role that prior theoretical knowledge can play in applying machine learning to biomechanics, especially given the often limited databases in this field due to high costs associated with experiments. However, this article should be viewed as a proof of concept study. Further studies based on much larger datasets ideally including also multiratio biaxial extension tests are required to confirm the results of this study and identify potential difficulties machine learning might face in soft tissue biomechanics. While such studies will require substantial effort, especially to collect sufficient amounts of experimental data, the promising results of this paper certainly give hope that they could have a transformative impact on soft tissue biomechanics.

## Data accessibility

The datasets analysed for this study were published before elsewhere.
